# Evaluation of the safety of high-dose *Lactobacillus gasseri* CP2305 supplementation in healthy adults: A randomized, double-blind, placebo-controlled study

**DOI:** 10.1016/j.toxrep.2026.102222

**Published:** 2026-02-13

**Authors:** Hajime Fuchida, Daisuke Sawada, Tatsuhiko Hirota, Hiroki Miyashita, Kazuhiko Takano, Koji Mochizuki

**Affiliations:** a Asahi Quality and Innovations Ltd., Department of Group Project Strategy, Moriya 302-0106, Japan; b Asahi Quality and Innovations, Ltd., Core Technology Laboratories, Moriya 302-0106, Japan; c Clinical Support Corporation, Clinical Trial Division Sapporo Branch, Hokkaido, Sapporo 060-0061, Japan; d Medical Corporation Hokubukai Utsukushigaoka Hospital, Hokkaido, Sapporo 004-0839, Japan

**Keywords:** L. gasseri CP2305, Safety, High-Dose

## Abstract

**Objective:**

*Lactobacillus gasseri CP2305 (L. gasseri CP2305)* has been reported to exert intestinal regulatory, anti-stress, and sleep-improving effects in healthy adults. This study aimed to evaluate the safety of an excessive intake of *L. gasseri* CP2305 in a randomized, double-blind, placebo-controlled, parallel-group trial.

**Methods:**

Among 95 participants who provided written informed consent, 44 who met the eligibility criteria were randomly assigned to either the *L. gasseri* CP2305 group (n = 22) or the placebo group (n = 22). All participants ingested 20 tablets of either *L. gasseri* CP2305 or placebo daily for 4 weeks. The *L. gasseri* CP2305 group consumed 1.0 × 10 ¹ ¹ cells of *L. gasseri* CP2305 cells per day. To assess the safety of CP2305 tablets, physical examinations, hematological and biochemical blood tests, urinalysis, recording of subjective symptoms, and physician interviews were conducted.

**Results:**

One participant was excluded from the analysis; thus, the final analysis included data from 22 participants in the *L. gasseri* CP2305 group and 21 participants in the placebo group. No adverse clinical findings were observed in either group.

**Conclusion:**

The results of this study demonstrate that no safety concerns were identified in the physical examinations, hematology, blood biochemistry, urinalysis, or subjective symptoms. These findings support the safety of *L. gasseri* CP2305 tablets even at high-dose intake (1.0 ×10 ¹¹ cells/day).

## Highlights

• A randomized, double-blind, placebo-controlled safety study was conducted in healthy adults.
• Participants consumed *Lactobacillus gasseri* CP2305 tablets at a dose up to ten times the recommended daily intake for 4 weeks.
• Safety was evaluated through physical examinations, hematology, blood biochemistry, urinalysis, and subjective symptoms.
• No safety concerns were identified during the study period.
• These findings confirm the safety of excessive intake of *L. gasseri* CP2305 tablets.

## 1 Introduction

Approximately 100 trillion bacteria and other microorganisms reside in the human intestines, forming diverse colonies known as the gut microbiota [1]. The composition and metabolism of the gut microbiota are influenced by dietary habits [2], stress [3], aging [4], and various other factors, and are also affected by several diseases [5], which can reduce quality of life. Therefore, improving the intestinal environment is essential for maintaining and promoting health. *Lactobacillus gasseri* CP2305, a lactic acid bacterium isolated from a healthy adult, has been the subject of numerous studies. It has been shown to exert intestinal regulatory effects that improve the intestinal environment, as well as anti-stress and sleep-improving effects.

The various effects, including intestinal regulatory effects in healthy individuals [6], improvements in stress and sleep among medical students [7], [8], enhanced quality of life and reduced irritable bowel syndrome (IBS) severity scores in patients with IBS [9], and alleviation of mild menopausal symptoms in women [10] were reported in human studies with this strain. Furthermore, in 2022, a systematic review and meta-analysis reported that daily intake of *L. gasseri* CP2305 significantly improved sleep quality in adults experiencing mild to moderate stress [11]. However, to date, no published studies have evaluated the safety of overdosing on this strain.

Therefore, the present study aimed to assess the safety of excessive intake of *L. gasseri* CP2305 through a 4-week intervention in healthy individuals and those prone to constipation, using a tablet-type food containing sterilized powder of the strain and a placebo food without the strain.

## 2 Materials and methods

### 2.1 Study design

A randomized, double-blind, placebo-controlled, parallel-group study was conducted at Utsukushigaoka Hospital (Hokubukai Medical Corporation, Sapporo, Japan) to evaluate the safety of excessive intake of *Lactobacillus gasseri* CP2305. The study protocol was approved by the Ethics Committee of Utsukushigaoka Hospital on February 25, 2016.

This study was conducted in accordance with the ethical principles outlined in the Declaration of Helsinki and the *Ethical Guidelines for Medical Research Involving Human Subjects* (Ministry of Education, Culture, Sports, Science and Technology, Japan, and Ministry of Health, Labour and Welfare, Japan). The study was performed in compliance with the approved protocol. The protocol was registered in the University Hospital Medical Information Network Clinical Trials Registry (UMIN-CTR) under the identifier UMIN000021511.

### 2.2 Subjects

The participants in this study were healthy Japanese adults.

**Inclusion criteria** were as follows:

(1) untreated Japanese men and women aged 20–70 years at the time of consent.
(2) individuals whom the principal investigator judged eligible for participation based on the results of a screening test.
(3) individuals who provided written, voluntary informed consent to participate in the study.

**Exclusion criteria** were as follows:

(1) individuals with a serious medical history that would make them unsuitable for participation.
(2) individuals with a history of digestive tract surgery, such as gastrectomy or intestinal resection (excluding appendectomy).
(3) individuals using medications for constipation or foods with equivalent effects (including “Food for Specified Health Uses”, “Foods with functional claim”, and dietary supplements).
(4) individuals with known allergies to lactic acid bacteria, milk, soy, citrus fruits, or related ingredients.
(5) women who intended to become pregnant during the study period, pregnant women (including those who might be pregnant), or breastfeeding women.
(6) individuals with excessive alcohol consumption or smoking habits.
(7) individuals with highly irregular eating habits (e.g., skipping meals three or more times per week).
(8) individuals engaged in night-shift work extending past midnight.
(9) individuals currently participating in other clinical trials or monitoring studies (for foods, pharmaceuticals, cosmetics, or topical products), or those planning to participate during the study period.
(10) individuals otherwise deemed unsuitable for participation by the principal investigator.

Participants were recruited through the clinical trial volunteer organization “Hokuto Unity Club” (operated by Utsukushigaoka Hospital) and Huma Co., Ltd. Before enrollment, all participants received a detailed explanation of the study using an information sheet approved by the Ethics Committee of Utsukushigaoka Hospital, and written informed consent was obtained. The intervention period was conducted from March to May 2016.

### 2.3 Sample size

The sample size, 22 participants per group, was determined referred to previous studies evaluating the safety of functional foods [12], [13], [14].

### 2.4 Enrollment, randomization, and blinding

A total of 95 individuals who provided written informed consent underwent screening, and 44 were enrolled in this study. Given that *Lactobacillus gasseri CP2305* has been reported to exert a regulatory effect on intestinal function, particularly in individuals with hard stools [6], the study population was stratified into healthy subjects and constipated subjects. The latter group was defined as individuals with a defecation frequency of fewer than four times per week, and included 16 participants

These 44 participants were randomly assigned to two groups by an individual not directly involved in the study. Random numbers were generated using Microsoft Excel to ensure that the average age of the two groups was approximately equal. Another independent individual then assigned the CP2305 tablets or placebo to the respective groups. The allocation information was sealed by each assignor, and the details of the assignment were kept concealed from all investigators and study staff until the code was broken after study completion.

### 2.5 Intervention

*Lactobacillus gasseri* strain CP2305, a probiotic strain isolated from a healthy human adult [7], was used in this study. A nonviable (heat-inactivated) CP2305 powder, containing at least 7.6 × 1011 cells per gram was obtained as previously described [6].

All participants were instructed to chew and ingest 20 study tablets of *L. gasseri* CP2305 or placebo daily for four weeks. In line with the actual usage of this product, no specific timing for intake was specified. If swallowing was difficult, they were permitted to take the tablets with an appropriate amount of water. The CP2305 tablets contained 3 % *L. gasseri* CP2305 powder, corresponding to 1.0 × 10 ¹ ¹ cells per 20 tablets per day (ten times the recommended daily intake). The placebo tablets did not contain *L. gasseri* CP2305 powder but instead contained an equivalent amount of dextrin. Both study tablets (CP2305 and placebo) weighed approximately 4.4 g per 20 tablets and were milky white in appearance.

### 2.6 Outcomes

The study schedule is shown in Table 1. Participants visited the hospital for screening, during which they underwent medical and physical examinations, hematological and biochemical blood tests, and urinalysis. They subsequently visited the hospital at weeks 0 (0 W), 2 (2 W), and 4 (4 W) during the intervention period, and again two weeks after the end of intake (6 W) for follow-up. At each visit, the same examinations were performed to collect safety data.

**Table 1:** Test schedule.

Participants were instructed to comply with the following eight rules throughout the study:

1. Participants were instructed to pay close attention to their personal health management and to avoid strenuous exercise or heavy physical labor. They were also asked to maintain their usual dietary and exercise habits as consistently as possible throughout the study period, relative to their pre-study routines.
2. The study tablets were to be consumed appropriately while maintaining regular lifestyle habits. In cases where medical treatment became necessary due to illness or other reasons, participants were instructed to contact the study staff and follow their guidance.
3. The use of any medications or foods that claim to regulate intestinal function was prohibited. However, if treatment became necessary due to an acute illness or other reasons, participants were to consult the study’s designated contact and adhere to the provided instructions.
4. Participants were asked to refrain from dieting, excessive alcohol consumption, and heavy smoking during the study period.
5. Strict adherence to the assigned visit schedule was required, and participants were instructed to carefully review the study schedule.
6. On the day before each hospital visit, participants were instructed to avoid high-fat meals and to abstain from alcohol consumption.
7. During each visit, participants were required to follow the instructions of the study physician, nurses, and research staff.
8. Participants were prohibited from sharing or disclosing any study materials or study tablets used in this study to third parties.

### 2.7 Primary outcomes

During the physical examinations, the subjects’ height, weight, body fat percentage, BMI, blood pressure, pulse rate, and body temperature were measured. Height was measured only at screening (SCR) to calculate BMI.

Hematological tests included WBC, RBC, Hb, Ht, and Plt. Blood biochemical tests included TP, Alb, T-Bil, AST (GOT), ALT (GPT), ALP, LDH, γ-GTP, T-Cho, HDL-Cho, LDL-Cho, TG, UA, BUN, CRE, Na, K, Cl, BS, IRI, and HbA1c. Both hematological and blood biochemical analyses were performed at Sapporo Clinical Laboratory Inc. (Sapporo, Japan).

Urinalysis included protein, glucose, urobilinogen, bilirubin, ketone bodies, occult blood, pH, and specific gravity. These analyses were also conducted at Sapporo Clinical Laboratory Inc. (Sapporo, Japan).

From the start of study tablets consumption until the post-observation period, the subjects kept a daily diary in which they recorded their daily routines (wake-up time, bedtime, and meal times), whether and when they had consumed the study tablets (and the reason if they did not), alcohol intake, use of health foods or medications, any subjective symptoms (e.g., dry cough, loss of appetite, gastric discomfort, abdominal pain, diarrhea, headache, and other related symptoms), and menstrual status. The subjects were instructed to bring their diaries to each hospital visit, where the entries were reviewed. In addition, at each visit, the subjects were examined and interviewed by a physician.

### 2.8 Statistical analysis

The mean and standard deviation of the data obtained at screening were calculated and used as background information.

Data collected at weeks 0 (0 W), 2 (2 W), 4 (4 W), and at post-observation (6 W) were used for analyses. Subjects who consumed less than 90 % of the study tablets Placebo during the 28-day period were excluded from the analysis. In addition, subjects deemed unsuitable for inclusion after the start of the study were also excluded. The mean and standard deviation were calculated for each data point, excluding dropouts. Paired *t*-tests were performed to compare within-group changes from baseline (0 W) to post-intake (2 W, 4 W, and 6 W). Unpaired *t*-tests were conducted to compare the CP2305 group and the placebo group at each visit. All statistical analyses were two-sided with a significance level of 5 %. No adjustment for multiple comparisons was applied. Statistical analyses were performed using Microsoft Excel (Microsoft Corp., Redmond, WA, USA).

## 3 Results

### 3.1 Setting analysis

Table 2 presents the background characteristics of the subjects (values at SCR). The subjects represented a broad range of age, with no apparent age-group bias. The subjects comprised equal numbers of men and women. No significant differences were observed between the groups in age or any other measured parameters.

**Table 2:** Subject background (values at screening).

	Items	Unit	Placebo group			CP2305 group		
Overall	Number	No.(male/female)	22(11/11)			22(11/11)		
	Age	years old	46.8	±	14.1	46.5	±	15.1
	Height	cm	164.05	±	10.24	162.22	±	8.56
	Body weight	kg	61.09	±	11.06	60.30	±	12.67
	BMI	m^2^/kg	22.57	±	2.57	22.71	±	3.31
	body fat percentage	%	26.19	±	7.23	26.07	±	5.94
Healthy subject	Number	No.(male/female)	14(7/7)			14(7/7)		
	Age	years old	45.1	±	15.5	45.1	±	16.5
	Height	cm	163.59	±	11.61	161.54	±	7.91
	Body weight	kg	61.00	±	12.46	59.91	±	13.83
	BMI	m^2^/kg	22.62	±	2.59	22.72	±	3.81
	body fat percentage	%	25.89	±	6.44	26.00	±	6.19
Constipated subject	Number	No.(male/female)	8(4/4)			8(4/4)		
	Age	years old	49.8	±	11.7	49.0	±	13.0
	Height	cm	164.85	±	7.93	163.41	±	10.06
	Body weight	kg	61.24	±	8.89	60.99	±	11.20
	BMI	m^2^/kg	22.49	±	2.72	22.70	±	2.42
	body fat percentage	%	26.73	±	8.91	26.19	±	5.88

All values except for the number of subjects are "mean ± standard deviation"

**Fig. 1:**
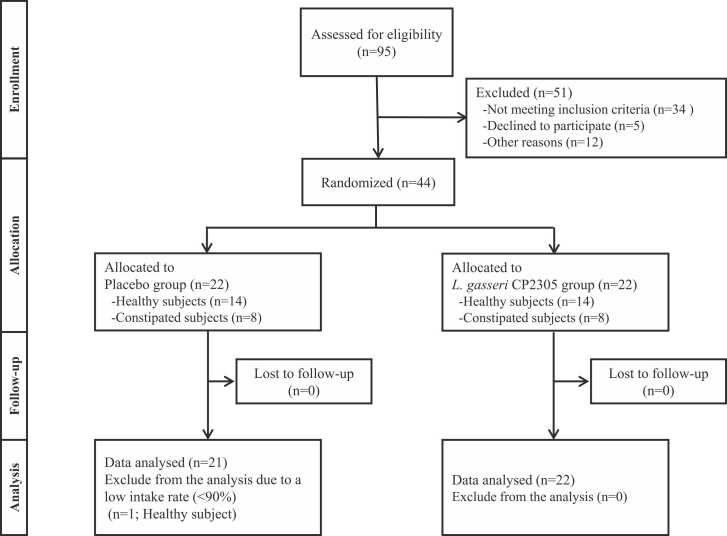
Flow diagram of participant screening, enrollment, and analysis.

All 44 enrolled subjects completed the study (Fig. 1). One subject (No. 104, male) had a study tablets intake rate of less than 90 % and was therefore excluded from the analysis. No other subjects violated the study protocol, and the final analysis was conducted on 43 subjects (Fig. 1).

### 3.2 Physical examination

The results of the physical examinations are shown in Table 3.

**Table 3:** Physical examinations.

				Intervention period																Post Observation				
	Items	reference		0 W					2 W						4 W					6 W				
		values†					BG					BA	BG					BA	BG				BA	BG
Overall	Body weight （kg）		Placebo	61.06	±	11.05			60.24	±	10.77	**			60.46	±	10.74	*		60.33	±	10.68	*	
			CP2305	61.00	±	12.89	NS		60.56	±	12.75	*	NS		60.67	±	12.73	NS	NS	60.65	±	12.76	NS	NS
	BMI （kg/m2）		Placebo	22.71	±	2.70			22.40	±	2.66	**			22.49	±	2.62	*		22.44	±	2.68	*	
			CP2305	23.00	±	3.40	NS		22.84	±	3.40	*	NS		22.88	±	3.36	NS	NS	22.86	±	3.35	NS	NS
	Body fat percentage （%）		Placebo	26.91	±	7.76			25.88	±	7.57	**			26.06	±	7.49	**		25.94	±	7.73	**	
			CP2305	26.53	±	6.31	NS		25.87	±	6.22	**	NS		25.81	±	6.42	**	NS	25.77	±	6.34	*	NS
	Systolic blood pressure （mmHg）	< 130	Placebo	119.5	±	16.3			118.5	±	15.3	NS			116.2	±	13.2	NS		116.3	±	12.6	NS	
			CP2305	120.7	±	18.8	NS		121.6	±	14.8	NS	NS		119.7	±	17.3	NS	NS	121.2	±	15.1	NS	NS
	Diastolic blood pressure （mmHg）	< 85	Placebo	69.7	±	13.0			73.2	±	15.6	NS			68.1	±	11.5	NS		66.9	±	9.1	NS	
			CP2305	70.1	±	12.0	NS		70.8	±	12.1	NS	NS		71.6	±	13.8	NS	NS	69.8	±	13.5	NS	NS
	Pulse rate （bpm）	< 100	Placebo	72.3	±	12.6			73.6	±	12.5	NS			72.1	±	13.5	NS		72.2	±	10.9	NS	
			CP2305	72.6	±	9.7	NS		73.3	±	10.5	NS	NS		72.4	±	8.3	NS	NS	71.7	±	9.0	NS	NS
	Body temperature （℃）		Placebo	36.34	±	0.39			36.30	±	0.48	NS			36.43	±	0.26	NS		36.50	±	0.32	NS	
			CP2305	36.40	±	0.31	NS		36.33	±	0.36	NS	NS		36.41	±	0.36	NS	NS	36.49	±	0.25	NS	NS
Healthy subject	Body weight （kg）		Placebo	60.78	±	12.56			59.69	±	12.26	**			59.97	±	12.25	*		59.83	±	12.21	*	
			CP2305	60.63	±	14.04	NS		60.17	±	14.06	*	NS		60.04	±	13.82	*	NS	59.99	±	13.74	NS	NS
	BMI （kg/m2）		Placebo	22.78	±	2.76			22.37	±	2.78	**			22.46	±	2.67	*		22.42	±	2.85	*	
			CP2305	23.01	±	3.87	NS		22.84	±	3.89	*	NS		22.81	±	3.83	*	NS	22.79	±	3.81	NS	NS
	Body fat percentage （%）		Placebo	26.60	±	7.38			25.35	±	6.99	*			25.74	±	6.90	*		25.89	±	7.10	*	
			CP2305	26.54	±	6.69	NS		25.91	±	6.55	*	NS		25.81	±	6.73	*	NS	25.58	±	6.89	*	NS
	Systolic blood pressure （mmHg）	< 130	Placebo	117.2	±	18.4			113.8	±	14.9	NS			115.2	±	12.3	NS		113.3	±	13.2	NS	
			CP2305	122.9	±	21.2	NS		125.8	±	15.1	NS	#		120.1	±	20.2	NS	NS	123.0	±	16.1	NS	NS
	Diastolic blood pressure （mmHg）	< 85	Placebo	66.9	±	13.0			66.5	±	11.2	NS			65.5	±	10.8	NS		64.5	±	9.0	NS	
			CP2305	70.5	±	13.5	NS		73.3	±	12.6	NS	NS		70.6	±	15.5	NS	NS	69.1	±	14.9	NS	NS
	Pulse rate （bpm）	< 100	Placebo	72.0	±	12.3			72.9	±	12.7	NS			73.5	±	16.1	NS		71.8	±	12.5	NS	
			CP2305	74.4	±	11.2	NS		76.4	±	11.4	NS	NS		72.8	±	6.7	NS	NS	72.6	±	10.1	NS	NS
	Body temperature （℃）		Placebo	36.38	±	0.45			36.30	±	0.50	NS			36.38	±	0.31	NS		36.48	±	0.31	NS	
			CP2305	36.35	±	0.33	NS		36.37	±	0.34	NS	NS		36.41	±	0.33	NS	NS	36.52	±	0.26	NS	NS
Constipated subject	Body weight （kg）		Placebo	61.51	±	8.81			61.14	±	8.53	NS			61.26	±	8.41	NS		61.14	±	8.33	NS	
			CP2305	61.66	±	11.47	NS		61.25	±	10.94	NS	NS		61.78	±	11.37	NS	NS	61.79	±	11.63	NS	NS
	BMI （kg/m2）		Placebo	22.60	±	2.77			22.46	±	2.65	NS			22.53	±	2.70	NS		22.46	±	2.59	NS	
			CP2305	22.96	±	2.64	NS		22.84	±	2.57	NS	NS		23.00	±	2.57	NS	NS	23.00	±	2.59	NS	NS
	Body fat percentage （%）		Placebo	27.43	±	8.84			26.73	±	8.88	NS			26.58	±	8.85	NS		26.03	±	9.18	**	
			CP2305	26.50	±	6.04	NS		25.80	±	6.02	NS	NS		25.81	±	6.29	NS	NS	26.10	±	5.68	NS	NS
	Systolic blood pressure （mmHg）	< 130	Placebo	123.1	±	12.6			126.1	±	13.5	NS			117.9	±	15.4	NS		121.1	±	10.7	NS	
			CP2305	116.9	±	14.2	NS		114.3	±	11.7	NS	NS		119.0	±	11.6	NS	NS	118.1	±	13.6	NS	NS
	Diastolic blood pressure （mmHg）	< 85	Placebo	74.1	±	12.5			84.1	±	16.0	*			72.4	±	12.0	NS		70.6	±	8.5	NS	
			CP2305	69.4	±	9.6	NS		66.4	±	10.6	NS	#		73.5	±	10.7	NS	NS	71.1	±	11.5	NS	NS
	Pulse rate （bpm）	< 100	Placebo	72.8	±	13.8			74.8	±	12.9	NS			69.8	±	8.1	NS		72.9	±	8.5	NS	
			CP2305	69.6	±	5.5	NS		67.9	±	6.1	NS	NS		71.6	±	11.1	NS	NS	70.0	±	6.7	NS	NS
	Body temperature （℃）		Placebo	36.28	±	0.29			36.29	±	0.47	NS			36.50	±	0.13	NS		36.53	±	0.34	NS	
			CP2305	36.50	±	0.28	NS		36.26	±	0.40	NS	NS		36.41	±	0.42	NS	NS	36.44	±	0.23	NS	NS

All values are "mean ± standard deviation"

**：Significant difference compared to 0 W（p < 0.01）

*：Significant difference compared to 0 W（p < 0.05）

##：Significant difference between the groups（p < 0.01）

#：Significant difference between the groups（p < 0.05）

NS：No significant difference

†：The reference values are those from the time of testing (2016).

BA：Significance test in comparison with 0 W

BG：Testing for significance between groups

In the overall analysis, body weight significantly decreased at 2 W, 4 W, and 6 W compared with 0 W in the placebo group (*p* < 0.001, *p* = 0.022, and *p* = 0.010, respectively). A significant decrement was also observed at 2 W in the CP2305 group (*p* = 0.027). BMI significantly decreased at 2 W, 4 W, and 6 W compared with 0 W in the placebo group (*p* < 0.001, *p* = 0.017, and *p* = 0.011, respectively) and at 2 W in the CP2305 group (*p* = 0.025). Body fat percentage significantly decreased at 2 W, 4 W, and 6 W in both groups (Placebo group: *p* = 0.010, 0.001, <0.001; CP2305 group: *p* = 0.002, 0.001, <0.001). Between the groups, no significant differences were observed for any of these parameters.

In the subgroup analysis of healthy subjects, weight loss was significantly greater at 2 W, 4 W, and 6 W compared with 0 W in the placebo group (*p* < 0.001, *p* = 0.041, *p* = 0.023). BMI significantly decreased at 2 W and 4 W in the CP2305 group (*p* = 0.025, *p* = 0.025), and at 2 W, 4 W, and 6 W in the placebo group (*p* < 0.001, *p* = 0.027, *p* = 0.020). Body fat percentage significantly decreased in both groups at 2 W, 4 W, and 6 W (Placebo group: *p* = 0.011, 0.012, 0.020; CP2305 group: *p* = 0.019, 0.015, 0.047). Furthermore, between-group comparison showed that systolic blood pressure at 2 W was significantly higher in the CP2305 group (*p* = 0.050), although values in both groups remained within the normal range.

In subjects prone to constipation, body fat percentage significantly decreased in the placebo group at 6 W (*p* = 0.010). Diastolic blood pressure significantly increased in the placebo group at 2 W (*p* = 0.011) and was significantly higher than that in the CP2305 group (*p* = 0.020).

### 3.3 Blood analysis

The results of the hematological tests are shown in Table 4.

**Table 4:** Hematological blood tests.

				Intervention period															Post Observation					
	Items	reference		0 W				2 W					4 W						6 W					
		values†					BG				BA	BG					BA	BG					BA	BG
Overall	White blood cells (*10^3^)	4.0～8.0	Placebo	6.73	±	1.62		6.60	±	1.78	NS		6.47	±	1.62		NS		6.95	±	2.43		NS	
			CP2305	6.16	±	1.17	NS	6.16	±	1.20	NS	NS	6.30	±	1.46		NS	NS	6.50	±	1.43		NS	NS
	Red blood cells (*10^4^)	M:410～540 F:380～500 (*10^4^)	Placebo	463.5	±	42.9		469.2	±	48.5	NS		462.4	±	46.2		NS		455.4	±	47.6		NS	
			CP2305	479.2	±	47.3	NS	480.7	±	53.7	NS	NS	477.0	±	57.4		NS	NS	481.5	±	49.8		NS	NS
	Hemoglobin (g/dL)	M:14.0～18.0 F:12.0～16.0	Placebo	14.11	±	1.13		14.35	±	1.34	NS		13.95	±	1.34		NS		13.77	±	1.32		*	
			CP2305	14.47	±	1.42	NS	14.55	±	1.54	NS	NS	14.30	±	1.64		NS	NS	14.53	±	1.55		NS	NS
	Hematocrit (%)	M:39.0～50.0 F:36.0～48.0	Placebo	42.54	±	2.87		42.90	±	3.69	NS		42.43	±	3.62		NS		42.31	±	3.39		NS	
			CP2305	43.82	±	3.57	NS	43.70	±	4.40	NS	NS	43.60	±	4.94		NS	NS	44.35	±	4.24		NS	NS
	Platelet (*10^4^)	12.0～40.0	Placebo	26.70	±	4.71		27.93	±	4.62	*		26.45	±	4.85		NS		27.70	±	6.62		NS	
			CP2305	24.89	±	3.60	NS	25.56	±	3.71	NS	NS	25.59	±	3.99		NS	NS	26.01	±	3.89		*	NS
Healthy subject	White blood cells (*10^3^)	4.0～8.0	Placebo	6.76	±	1.38		6.41	±	1.35	NS		6.21	±	1.44		*		6.76	±	1.35		NS	
			CP2305	6.14	±	1.14	NS	6.35	±	1.18	NS	NS	6.27	±	1.53		NS	NS	6.28	±	0.99		NS	NS
	Red blood cells (*10^4^)	M:410～540 F:380～500 (*10^4^)	Placebo	459.4	±	49.5		467.5	±	52.3	NS		462.4	±	49.8		NS		455.5	±	56.1		NS	
			CP2305	490.2	±	50.1	NS	489.1	±	52.6	NS	NS	486.6	±	58.2		NS	NS	483.3	±	52.4		NS	NS
	Hemoglobin (g/dL)	M:14.0～18.0 F:12.0～16.0	Placebo	14.19	±	1.27		14.46	±	1.31	NS		14.15	±	1.28		NS		13.92	±	1.45		NS	
			CP2305	14.70	±	1.63	NS	14.74	±	1.60	NS	NS	14.54	±	1.77		NS	NS	14.47	±	1.71		NS	NS
	Hematocrit (%)	M:39.0～50.0 F:36.0～48.0	Placebo	42.49	±	3.18		43.10	±	3.49	NS		42.48	±	2.99		NS		42.35	±	3.47		NS	
			CP2305	44.55	±	4.04	NS	44.08	±	4.67	NS	NS	44.31	±	5.28		NS	NS	44.34	±	4.64		NS	NS
	Platelet (*10^4^)	12.0～40.0	Placebo	26.35	±	4.80		26.97	±	2.89	NS		25.53	±	3.86		NS		26.31	±	3.88		NS	
			CP2305	24.64	±	4.10	NS	25.21	±	4.08	NS	NS	25.05	±	4.58		NS	NS	24.90	±	4.09		NS	NS
Constipated subject	White blood cells (*10^3^)	4.0～8.0	Placebo	6.68	±	2.06		6.90	±	2.40	NS		6.90	±	1.91		NS		7.26	±	3.68		NS	
			CP2305	6.21	±	1.31	NS	5.84	±	1.22	NS	NS	6.36	±	1.45		NS	NS	6.90	±	2.00		NS	NS
	Red blood cells (*10^4^)	M:410～540 F:380～500 (*10^4^)	Placebo	470.1	±	31.2		472.0	±	44.9	NS		462.4	±	43.1		NS		455.1	±	32.7		NS	
			CP2305	460.0	±	37.2	NS	466.1	±	55.8	NS	NS	460.0	±	55.5		NS	NS	478.5	±	48.4		*	NS
	Hemoglobin (g/dL)	M:14.0～18.0 F:12.0～16.0	Placebo	13.98	±	0.93		14.18	±	1.48	NS		13.63	±	1.45		NS		13.54	±	1.12		NS	
			CP2305	14.08	±	0.89	NS	14.20	±	1.45	NS	NS	13.89	±	1.38		NS	NS	14.64	±	1.31		*	NS
	Hematocrit (%)	M:39.0～50.0 F:36.0～48.0	Placebo	42.61	±	2.47		42.56	±	4.23	NS		42.36	±	4.70		NS		42.25	±	3.50		NS	
			CP2305	42.54	±	2.20	NS	43.04	±	4.10	NS	NS	42.36	±	4.32		NS	NS	44.36	±	3.75		*	NS
	Platelet (*10^4^)	12.0～40.0	Placebo	27.28	±	4.83		29.49	±	6.50	*		27.95	±	6.11		NS		29.96	±	9.49		NS	
			CP2305	25.33	±	2.70	NS	26.16	±	3.11	NS	NS	26.54	±	2.69		*	NS	27.96	±	2.74		**	NS

All values are "mean ± standard deviation"

**：Significant difference compared to 0 W（p < 0.01）

*：Significant difference compared to 0 W（p < 0.05）

##：Significant difference between the groups（p < 0.01）

#：Significant difference between the groups（p < 0.05）

NS：No significant difference

†：The reference values are those from the time of testing (2016).

BA：Significance test in comparison with 0 W

BG：Testing for significance between groups

In the analysis of all participants, significant changes were observed in several parameters compared with baseline; however, no significant differences were found between the groups. Similar trends were observed in both the healthy subgroup and the constipation-prone subgroup.

The results of the blood biochemical tests are presented in Table 5.

**Table 5:** Biochemical blood tests.

			Intervention period															Post Observation				
	Items	reference	Group	0 W				2 W					4 W					6 W				
		values†					BG				BA	BG				BA	BG				BA	BG
Overall	Total protein (g/dL)	6.7～8.3	Placebo	7.25	±	0.30		7.24	±	0.40	NS		7.29	±	0.36	NS		7.23	±	0.40	NS	
			CP2305	7.24	±	0.38	NS	7.19	±	0.38	NS	NS	7.29	±	0.40	NS	NS	7.33	±	0.34	NS	NS
	Albumin (g/dL)	3.8～5.3	Placebo	4.60	±	0.20		4.60	±	0.26	NS		4.63	±	0.26	NS		4.58	±	0.25	NS	
			CP2305	4.66	±	0.29	NS	4.61	±	0.25	NS	NS	4.69	±	0.28	NS	NS	4.65	±	0.28	NS	NS
	Total bilirubin (mg/dL)	0.2～1.2	Placebo	0.70	±	0.39		0.68	±	0.62	NS		0.64	±	0.27	NS		0.60	±	0.29	*	
			CP2305	0.67	±	0.21	NS	0.66	±	0.26	NS	NS	0.66	±	0.24	NS	NS	0.63	±	0.21	NS	NS
	ＡＳＴ（ＧＯＴ） (U/L)	8～38	Placebo	22.7	±	8.0		20.7	±	4.9	NS		22.2	±	4.6	NS		22.1	±	4.9	NS	
			CP2305	19.5	±	3.8	NS	19.3	±	3.3	NS	NS	20.8	±	5.4	*	NS	20.0	±	4.7	NS	NS
	ＡＬＴ（ＧＰＴ） (U/L)	4～44	Placebo	23.4	±	19.4		19.7	±	10.5	NS		22.0	±	13.2	NS		22.0	±	13.1	NS	
			CP2305	17.1	±	5.3	NS	18.0	±	5.8	NS	NS	18.4	±	8.0	NS	NS	18.5	±	6.6	NS	NS
	ＡＬＰ (U/L)	105～330	Placebo	196.2	±	51.9		192.0	±	47.4	NS		195.3	±	45.5	NS		203.0	±	62.2	NS	
			CP2305	219.4	±	56.9	NS	208.3	±	49.0	NS	NS	219.7	±	56.1	NS	NS	223.2	±	63.0	NS	NS
	ＬＤ（ＬＤＨ） (U/L)	120～245	Placebo	180.5	±	20.3		182.2	±	21.9	NS		191.9	±	18.4	**		190.4	±	23.0	**	
			CP2305	175.2	±	20.4	NS	175.2	±	18.5	NS	NS	185.2	±	23.5	**	NS	183.9	±	21.4	*	NS
	γ－ＧＴ (U/L)	M:≦ 80以下	Placebo	32.2	±	23.4		29.7	±	19.5	NS		30.1	±	20.4	NS		37.5	±	49.7	NS	
		F:≦ 30以下	CP2305	23.0	±	9.1	NS	23.3	±	9.9	NS	NS	24.2	±	9.9	NS	NS	24.7	±	11.0	NS	NS
	Total cholestesterol (mg/dL)	130～220	Placebo	214.0	±	34.5		211.6	±	36.2	NS		216.4	±	37.8	NS		209.2	±	35.2	NS	
			CP2305	214.7	±	37.5	NS	206.6	±	35.1	NS	NS	209.5	±	38.5	NS	NS	208.5	±	40.5	NS	NS
	ＨＤＬ cholestesterol (mg/dL)	M:40～90	Placebo	63.5	±	16.2		61.5	±	14.7	NS		63.0	±	15.9	NS		61.4	±	14.6	NS	
		F:40～100	CP2305	63.5	±	15.5	NS	60.2	±	14.1	**	NS	60.8	±	14.5	*	NS	60.1	±	16.9	*	NS
	ＬＤＬ cholestesterol (mg/dL)	70～139	Placebo	135.4	±	35.8		133.8	±	39.0	NS		136.2	±	38.4	NS		132.5	±	34.9	NS	
			CP2305	135.0	±	36.7	NS	127.2	±	34.8	*	NS	129.0	±	37.4	NS	NS	129.2	±	37.0	NS	NS
	Triglyceride (mg/dL)	30～149	Placebo	100.7	±	48.2		88.6	±	37.2	NS		99.7	±	72.1	NS		85.6	±	30.5	*	
			CP2305	103.1	±	70.5	NS	107.2	±	87.5	NS	NS	103.5	±	63.6	NS	NS	108.4	±	66.2	NS	NS
	Urin acid (mg/dL)	2.0～7.0	Placebo	5.31	±	1.18		5.19	±	1.08	NS		5.03	±	1.16	**		5.37	±	1.15	NS	
			CP2305	4.99	±	1.34	NS	4.87	±	1.10	NS	NS	4.86	±	1.11	NS	NS	5.02	±	1.01	NS	NS
	Urea nitrogen (mg/dL)	8.0～20.0	Placebo	13.25	±	3.70		13.19	±	3.24	NS		13.18	±	2.78	NS		13.69	±	2.68	NS	
			CP2305	13.05	±	2.57	NS	12.60	±	3.23	NS	NS	12.35	±	2.68	NS	NS	13.08	±	2.79	NS	NS
	Creatinine (mg/dL)	M:0.66～1.11	Placebo	0.810	±	0.122		0.788	±	0.110	NS		0.780	±	0.139	**		0.808	±	0.141	NS	
		F:0.50～0.86	CP2305	0.812	±	0.109	NS	0.780	±	0.096	**	NS	0.793	±	0.130	NS	NS	0.790	±	0.104	*	NS
	Ｎａ (mEq/L)	137～147	Placebo	141.1	±	1.2		141.8	±	1.7	NS		141.3	±	2.0	NS		141.4	±	1.8	NS	
			CP2305	141.0	±	1.3	NS	141.8	±	1.7	*	NS	142.0	±	1.4	**	NS	141.3	±	1.6	NS	NS
	Ｋ (mEq/L)	3.5～5.0	Placebo	4.13	±	0.35		4.08	±	0.34	NS		4.05	±	0.39	NS		3.77	±	0.33	**	
			CP2305	4.18	±	0.28	NS	4.15	±	0.26	NS	NS	3.92	±	0.32	**	NS	4.02	±	0.42	NS	#
	Ｃｌ (mEq/L)	98～108	Placebo	101.6	±	1.4		102.0	±	1.7	NS		101.9	±	2.0	NS		101.8	±	1.5	NS	
			CP2305	101.8	±	1.3	NS	102.2	±	1.9	NS	NS	102.1	±	1.6	NS	NS	101.5	±	1.9	NS	NS
	Blood glucose level (mg/dL)	70～109	Placebo	86.1	±	8.9		89.6	±	8.7	*		91.4	±	9.9	*		89.0	±	7.8	*	
			CP2305	83.5	±	7.9	NS	85.6	±	7.2	*	NS	87.3	±	8.5	*	NS	85.0	±	7.0	NS	NS
	Insulin (μU/mL)	1.1～17.0	Placebo	7.04	±	13.60		6.47	±	7.74	NS		7.59	±	13.33	NS		5.80	±	7.34	NS	
			CP2305	4.53	±	3.54	NS	4.19	±	3.19	NS	NS	4.34	±	3.20	NS	NS	5.17	±	4.07	NS	NS
	ＨｂＡ１ｃ (%)	4.6～6.2	Placebo	5.25	±	0.29		5.33	±	0.27	**		5.32	±	0.27	*		5.29	±	0.29	NS	
			CP2305	5.25	±	0.39	NS	5.27	±	0.37	NS	NS	5.26	±	0.38	NS	NS	5.21	±	0.38	NS	NS
		values†					BG				BA	BG				BA	BG				BA	BG
Healthy subject	Total protein (g/dL)	6.7～8.3	Placebo	7.15	±	0.29		7.13	±	0.39	NS		7.14	±	0.31	NS		7.08	±	0.37	NS	
			CP2305	7.26	±	0.41	NS	7.24	±	0.34	NS	NS	7.32	±	0.38	NS	NS	7.29	±	0.34	NS	NS
	Albumin (g/dL)	3.8～5.3	Placebo	4.62	±	0.23		4.61	±	0.27	NS		4.63	±	0.25	NS		4.58	±	0.19	NS	
			CP2305	4.69	±	0.31	NS	4.61	±	0.23	NS	NS	4.68	±	0.29	NS	NS	4.59	±	0.30	*	NS
	Total bilirubin (mg/dL)	0.2～1.2	Placebo	0.79	±	0.46		0.74	±	0.78	NS		0.68	±	0.31	NS		0.63	±	0.33	*	
			CP2305	0.69	±	0.24	NS	0.66	±	0.30	NS	NS	0.70	±	0.24	NS	NS	0.65	±	0.22	NS	NS
	ＡＳＴ（ＧＯＴ） (U/L)	8～38	Placebo	21.8	±	7.1		20.1	±	5.1	NS		22.2	±	5.1	NS		21.8	±	4.9	NS	
			CP2305	19.9	±	4.6	NS	19.0	±	3.3	NS	NS	21.3	±	6.4	NS	NS	20.1	±	5.4	NS	NS
	ＡＬＴ（ＧＰＴ） (U/L)	4～44	Placebo	22.2	±	18.2		18.8	±	11.7	NS		22.6	±	15.7	NS		21.1	±	13.7	NS	
			CP2305	17.8	±	5.9	NS	17.9	±	6.3	NS	NS	19.9	±	9.2	NS	NS	19.1	±	7.3	NS	NS
	ＡＬＰ (U/L)	105～330	Placebo	190.0	±	47.6		186.6	±	47.4	NS		189.5	±	46.8	NS		184.0	±	44.0	NS	
			CP2305	209.8	±	45.7	NS	203.4	±	41.4	NS	NS	211.6	±	45.3	NS	NS	211.1	±	43.9	NS	NS
	ＬＤ（ＬＤＨ） (U/L)	120～245	Placebo	181.7	±	19.8		182.5	±	18.1	NS		191.5	±	13.5	**		189.3	±	19.7	NS	
			CP2305	174.1	±	20.9	NS	175.5	±	17.4	NS	NS	186.1	±	24.7	**	NS	182.4	±	24.0	*	NS
	γ－ＧＴ (U/L)	M:≦ 80	Placebo	31.7	±	24.1		27.8	±	18.0	NS		28.4	±	18.5	NS		28.7	±	18.5	NS	
		F:≦ 30	CP2305	23.4	±	10.2	NS	22.4	±	8.7	NS	NS	24.1	±	9.1	NS	NS	23.2	±	10.1	NS	NS
	Total cholestesterol (mg/dL)	130～220	Placebo	219.5	±	33.8		214.1	±	34.7	NS		218.8	±	37.0	NS		211.2	±	33.9	NS	
			CP2305	217.9	±	35.4	NS	207.4	±	27.6	NS	NS	211.1	±	34.8	NS	NS	202.6	±	38.7	*	NS
	ＨＤＬ cholestesterol (mg/dL)	M:40～90	Placebo	67.8	±	16.6		65.8	±	16.1	NS		66.5	±	17.8	NS		64.7	±	15.3	NS	
		F:40～100	CP2305	65.1	±	13.9	NS	61.5	±	10.9	*	NS	62.1	±	11.4	*	NS	60.3	±	13.7	*	NS
	ＬＤＬ cholestesterol (mg/dL)	70～139	Placebo	138.2	±	34.2		133.2	±	35.8	NS		136.6	±	34.7	NS		131.8	±	33.2	NS	
			CP2305	136.9	±	34.1	NS	130.2	±	29.9	NS	NS	130.6	±	34.0	NS	NS	124.5	±	36.0	*	NS
	Triglyceride (mg/dL)	30～149	Placebo	89.4	±	42.8		80.4	±	38.7	NS		100.4	±	90.3	NS		81.8	±	33.0	NS	
			CP2305	109.4	±	72.4	NS	95.6	±	57.5	NS	NS	95.9	±	57.5	NS	NS	98.4	±	55.7	NS	NS
	Urin acid (mg/dL)	2.0～7.0	Placebo	5.43	±	1.29		5.18	±	1.25	NS		5.14	±	1.31	*		5.46	±	1.29	NS	
			CP2305	5.06	±	1.28	NS	4.96	±	0.82	NS	NS	4.99	±	0.86	NS	NS	5.06	±	0.86	NS	NS
	Urea nitrogen (mg/dL)	8.0～20.0	Placebo	13.14	±	4.34		13.15	±	3.34	NS		13.48	±	3.14	NS		13.72	±	3.01	NS	
			CP2305	13.30	±	2.21	NS	12.86	±	3.33	NS	NS	12.64	±	2.16	NS	NS	13.64	±	3.09	NS	NS
	Creatinine (mg/dL)	M:0.66～1.11	Placebo	0.815	±	0.127		0.800	±	0.109	NS		0.795	±	0.141	NS		0.813	±	0.142	NS	
		F:0.50～0.86	CP2305	0.793	±	0.101	NS	0.763	±	0.078	*	NS	0.778	±	0.123	NS	NS	0.774	±	0.098	NS	NS
	Ｎａ (mEq/L)	137～147	Placebo	141.2	±	1.1		141.3	±	1.3	NS		140.8	±	1.9	NS		141.5	±	1.6	NS	
			CP2305	141.2	±	0.9	NS	142.0	±	1.4	*	NS	142.1	±	1.4	**	NS	141.4	±	1.4	NS	NS
	Ｋ (mEq/L)	3.5～5.0	Placebo	4.02	±	0.27		3.96	±	0.21	NS		3.95	±	0.30	NS		3.64	±	0.28	**	
			CP2305	4.16	±	0.31	NS	4.09	±	0.25	NS	NS	4.01	±	0.36	NS	NS	3.95	±	0.37	NS	#
	Ｃｌ (mEq/L)	98～108	Placebo	101.5	±	1.2		101.4	±	1.6	NS		101.5	±	2.2	NS		101.8	±	1.6	NS	
			CP2305	102.2	±	1.1	NS	102.6	±	1.5	NS	#	102.4	±	1.7	NS	NS	102.1	±	1.7	NS	NS
	Blood glucose level (mg/dL)	70～109	Placebo	84.1	±	6.8		88.6	±	9.8	*		91.2	±	11.7	*		86.8	±	7.2	**	
			CP2305	83.4	±	7.0	NS	84.6	±	6.6	NS	NS	86.0	±	7.4	*	NS	83.8	±	6.4	NS	NS
	Insulin (μU/mL)	1.1～17.0	Placebo	3.95	±	1.85		5.09	±	3.23	NS		9.57	±	16.85	NS		4.42	±	1.95	NS	
			CP2305	4.44	±	2.78	NS	4.90	±	3.79	NS	NS	4.44	±	3.52	NS	NS	5.71	±	4.68	NS	NS
	ＨｂＡ１ｃ (%)	4.6～6.2	Placebo	5.18	±	0.27		5.28	±	0.24	*		5.28	±	0.22	*		5.26	±	0.24	NS	
			CP2305	5.26	±	0.41	NS	5.29	±	0.39	NS	NS	5.29	±	0.39	NS	NS	5.23	±	0.41	NS	NS
Constipated subject	Total protein (g/dL)	6.7～8.3	Placebo	7.43	±	0.24		7.43	±	0.37	NS		7.53	±	0.31	NS		7.49	±	0.30	NS	
			CP2305	7.20	±	0.35	NS	7.11	±	0.45	NS	NS	7.23	±	0.46	NS	NS	7.41	±	0.34	NS	NS
	Albumin (g/dL)	3.8～5.3	Placebo	4.59	±	0.16		4.59	±	0.28	NS		4.63	±	0.31	NS		4.56	±	0.35	NS	
			CP2305	4.61	±	0.25	NS	4.61	±	0.31	NS	NS	4.70	±	0.28	NS	NS	4.75	±	0.23	NS	NS
	Total bilirubin (mg/dL)	0.2～1.2	Placebo	0.56	±	0.14		0.59	±	0.16	NS		0.59	±	0.21	NS		0.54	±	0.19	NS	
			CP2305	0.64	±	0.15	NS	0.66	±	0.21	NS	NS	0.59	±	0.23	NS	NS	0.60	±	0.20	NS	NS
	ＡＳＴ（ＧＯＴ） (U/L)	8～38	Placebo	24.1	±	9.7		21.6	±	4.5	NS		22.1	±	3.8	NS		22.8	±	5.0	NS	
			CP2305	18.9	±	2.0	NS	19.9	±	3.4	NS	NS	20.0	±	3.2	NS	NS	19.9	±	3.7	NS	NS
	ＡＬＴ（ＧＰＴ） (U/L)	4～44	Placebo	25.4	±	22.4		21.0	±	8.9	NS		21.1	±	8.6	NS		23.4	±	12.9	NS	
			CP2305	16.0	±	4.1	NS	18.1	±	5.1	NS	NS	15.9	±	5.0	NS	NS	17.6	±	5.4	NS	NS
	ＡＬＰ (U/L)	105～330	Placebo	206.3	±	60.2		200.6	±	49.2	NS		204.6	±	44.7	NS		233.9	±	77.4	NS	
			CP2305	236.1	±	72.9	NS	216.8	±	62.3	NS	NS	233.8	±	72.6	NS	NS	244.5	±	86.7	NS	NS
	ＬＤ（ＬＤＨ） (U/L)	120～245	Placebo	178.5	±	22.4		181.9	±	28.4	NS		192.5	±	25.6	*		192.3	±	29.0	**	
			CP2305	177.1	±	20.7	NS	174.8	±	21.7	NS	NS	183.5	±	22.9	NS	NS	186.5	±	17.1	NS	NS
	γ－ＧＴ (U/L)	M:≦ 80	Placebo	33.1	±	23.8		32.8	±	22.6	NS		33.0	±	24.1	NS		51.9	±	78.1	NS	
		F:≦ 30	CP2305	22.3	±	7.7	NS	24.9	±	12.3	NS	NS	24.3	±	11.9	NS	NS	27.4	±	12.7	NS	NS
	Total cholestesterol (mg/dL)	130～220	Placebo	205.0	±	36.0		207.6	±	40.6	NS		212.5	±	41.4	NS		205.9	±	39.3	NS	
			CP2305	209.1	±	42.8	NS	205.4	±	47.6	NS	NS	206.6	±	46.8	NS	NS	218.8	±	44.2	NS	NS
	ＨＤＬ cholestesterol (mg/dL)	M:40～90	Placebo	56.4	±	13.4		54.5	±	9.1	NS		57.3	±	10.7	NS		56.0	±	12.3	NS	
		F:40～100	CP2305	60.9	±	18.6	NS	58.0	±	19.2	NS	NS	58.6	±	19.4	NS	NS	59.8	±	22.6	NS	NS
	ＬＤＬ cholestesterol (mg/dL)	70～139	Placebo	131.0	±	40.2		134.6	±	46.4	NS		135.6	±	46.4	NS		133.6	±	39.9	NS	
			CP2305	131.5	±	43.0	NS	121.9	±	43.9	NS	NS	126.3	±	45.0	NS	NS	137.4	±	39.9	NS	NS
	Triglyceride (mg/dL)	30～149	Placebo	119.0	±	53.6		101.9	±	32.8	NS		98.5	±	29.3	NS		91.9	±	26.8	NS	
			CP2305	92.0	±	70.4	NS	127.6	±	126.8	NS	NS	116.9	±	75.3	*	NS	125.8	±	82.7	**	NS
	Urin acid (mg/dL)	2.0～7.0	Placebo	5.11	±	1.04		5.19	±	0.83	NS		4.86	±	0.92	NS		5.23	±	0.94	NS	
			CP2305	4.88	±	1.53	NS	4.70	±	1.52	NS	NS	4.63	±	1.50	NS	NS	4.94	±	1.29	NS	NS
	Urea nitrogen (mg/dL)	8.0～20.0	Placebo	13.44	±	2.62		13.25	±	3.28	NS		12.69	±	2.17	NS		13.63	±	2.23	NS	
			CP2305	12.61	±	3.22	NS	12.13	±	3.22	NS	NS	11.86	±	3.52	NS	NS	12.09	±	1.98	NS	NS
	Creatinine (mg/dL)	M:0.66～1.11	Placebo	0.804	±	0.123		0.768	±	0.116	**		0.755	±	0.143	*		0.799	±	0.148	NS	
		F:0.50～0.86	CP2305	0.846	±	0.122	NS	0.811	±	0.120	**	NS	0.819	±	0.147	NS	NS	0.819	±	0.115	NS	NS
	Ｎａ (mEq/L)	137～147	Placebo	141.1	±	1.6		142.6	±	2.0	*		142.0	±	2.1	NS		141.4	±	2.2	NS	
			CP2305	140.6	±	1.8	NS	141.4	±	2.2	NS	NS	141.6	±	1.4	NS	NS	141.1	±	2.0	NS	NS
	Ｋ (mEq/L)	3.5～5.0	Placebo	4.31	±	0.40		4.28	±	0.43	NS		4.21	±	0.49	NS		3.98	±	0.32	*	
			CP2305	4.20	±	0.23	NS	4.24	±	0.27	NS	NS	3.78	±	0.16	**	#	4.15	±	0.49	NS	NS
	Ｃｌ (mEq/L)	98～108	Placebo	101.6	±	1.8		102.9	±	1.4	*		102.4	±	1.7	NS		101.9	±	1.2	NS	
			CP2305	101.1	±	1.2	NS	101.5	±	2.3	NS	NS	101.8	±	1.5	NS	NS	100.3	±	1.7	NS	#
	Blood glucose level (mg/dL)	70～109	Placebo	89.5	±	11.2		91.3	±	6.8	NS		91.8	±	6.9	NS		92.5	±	7.7	NS	
			CP2305	83.8	±	9.7	NS	87.3	±	8.4	NS	NS	89.6	±	10.3	NS	NS	87.0	±	7.9	NS	NS
	Insulin (μU/mL)	1.1～17.0	Placebo	12.08	±	21.81		8.71	±	11.99	NS		4.36	±	1.29	NS		8.04	±	11.76	NS	
			CP2305	4.69	±	4.81	NS	2.94	±	0.99	NS	NS	4.18	±	2.79	NS	NS	4.23	±	2.70	NS	NS
	ＨｂＡ１ｃ (%)	4.6～6.2	Placebo	5.38	±	0.31		5.43	±	0.32	NS		5.39	±	0.34	NS		5.33	±	0.38	NS	
			CP2305	5.23	±	0.39	NS	5.24	±	0.36	NS	NS	5.23	±	0.37	NS	NS	5.19	±	0.36	NS	NS

All values are "mean ± standard deviation"

**：Significant difference compared to 0 W（p < 0.01）

*：Significant difference compared to 0 W（p < 0.05）

##：Significant difference between the groups（p < 0.01）

#：Significant difference between the groups（p < 0.05）

NS：No significant difference

†：The reference values are those from the time of testing (2016).

BA：Significance test in comparison with 0 W

BG：Testing for significance between groups

In the analysis of all participants, significant changes were observed in several parameters compared with baseline, and a significant between-group difference was found for potassium at 6 W (*p* = 0.032). Among healthy subjects, significant changes were also observed in several parameters compared with baseline, with a significant between-group difference in potassium at 6 W (*p* = 0.021). Similarly, in the constipation-prone subgroup, significant within-group changes were observed in multiple parameters, and significant between-group differences were detected for potassium at 4 W (*p* = 0.029) and for chloride at 6 W (*p* = 0.045).

### 3.4 Urinalysis

No significant differences were observed between the groups in urinalysis results (Table 6).

**Table 6:** Urinalysis.

								Intervention period										Post Observation				
	Items	reference	Group	0 W				2 W					4 W					6 W				
		values†					BG				BA	BG				BA	BG				BA	BG
Overall	urine protein	(-)	Placebo	(-):19 (+-):2				(-):17 (+-):2 (+):2					(-):20 (+-):1					(-):18 (+-):2 (+):1				
			CP2305	(-):18 (+-):3 (+):1				(-):18 (+-):4					(-):22					(-):21 (+-):1				
	Urine glucose (qualitative)	(-)	Placebo	(-):21				(-):21					(-):21					(-):21				
			CP2305	(-):22				(-):22					(-):22					(-):22				
	Urobilinogen	(+-)	Placebo	(+-):21				(+-):21					(+-):21					(+-):21				
			CP2305	(+-):22				(+-):22					(+-):22					(+-):21 (+):1				
	Urinary bilirubin	(-)	Placebo	(-):21				(-):21					(-):21					(-):21				
			CP2305	(-):22				(-):22					(-):22					(-):22				
	Urinary ketones	(-)	Placebo	(-):21				(-):21					(-):21					(-):19 (+):2				
			CP2305	(-):20 (+):2				(-):22					(-):22					(-):22				
	Urine occult blood	(-)	Placebo	(-):19 (+-):1 (++):1				(-):17 (+-):3 (+):1					(-):15 (+-):3 (+):2 (++):1					(-):20 (+-):1				
			CP2305	(-):17 (+-):2 (++):1 (+++):2				(-):18 (+-):2 (+):2					(-):19 (++):1 (+++):2					(-):21 (+):1				
	Urine ｐＨ	5.0～7.5	Placebo	6.17	±	0.80		6.02	±	0.66	NS		6.36	±	0.81	NS		6.10	±	0.74	NS	
			CP2305	6.52	±	0.91	NS	6.05	±	0.79	NS	NS	6.02	±	0.63	*	NS	6.16	±	0.71	NS	NS
	Urine specific gravity	1.006～1.030	Placebo	1.0153	±	0.0081		1.0160	±	0.0084	NS		1.0140	±	0.0075	NS		1.0174	±	0.0081	NS	
			CP2305	1.0130	±	0.0082	NS	1.0165	±	0.0086	*	NS	1.0145	±	0.0097	NS	NS	1.0157	±	0.0086	NS	NS
Healthy subject	urine protein	(-)	Placebo	(-):12 (+-):1				(-):11 (+-):1 (+):1					(-):12 (+-):1					(-):11 (+-):2				
			CP2305	(-):12 (+-):2				(-):12 (+-):2					(-):14					(-):13 (+-):1				
	Urine glucose (qualitative)	(-)	Placebo	(-):13				(-):13					(-):13					(-):13				
			CP2305	(-):14				(-):14					(-):14					(-):14				
	Urobilinogen	(+-)	Placebo	(+-):13				(+-):13					(+-):13					(+-):13				
			CP2305	(+-):14				(+-):14					(+-):14					(+-):13 (+):1				
	Urinary bilirubin	(-)	Placebo	(-):13				(-):13					(-):13					(-):13				
			CP2305	(-):14				(-):14					(-):14					(-):14				
	Urinary ketones	(-)	Placebo	(-):13				(-):13					(-):13					(-):12 (+):1				
			CP2305	(-):13 (+):1				(-):14					(-):14					(-):14				
	Urine occult blood	(-)	Placebo	(-):12 (+-):1				(-):12 (+-):1					(-):9 (+-):1 (+):2 (++):1					(-):12 (+-):1				
			CP2305	(-):11 (+-):1 (++):1 (+++):1				(-):13 (+):1					(-):12 (++):1 (+++):1					(-):13 (+):1				
	Urine ｐＨ	5.0～7.5	Placebo	6.19	±	0.90		5.96	±	0.48	NS		6.35	±	0.92	NS		5.96	±	0.69	NS	
			CP2305	6.57	±	1.02	NS	5.86	±	0.74	*	NS	6.00	±	0.71	NS	NS	6.14	±	0.74	NS	NS
	Urine specific gravity	1.006～1.030	Placebo	1.0154	±	0.0090		1.0183	±	0.0080	NS		1.0147	±	0.0083	NS		1.0188	±	0.0083	NS	
			CP2305	1.0129	±	0.0078	NS	1.0181	±	0.0086	*	NS	1.0168	±	0.0104	*	NS	1.0179	±	0.0090	NS	NS
Constipated subject	urine protein	(-)	Placebo	(-):7 (+-):1				(-):6 (+-):1 (+):1					(-):8					(-):7 (+):1				
			CP2305	(-):6 (+-):1 (+):1				(-):6 (+-):2					(-):8					(-):8				
	Urine glucose (qualitative)	(-)	Placebo	(-):8				(-):8					(-):8					(-):8				
			CP2305	(-):8				(-):8					(-):8					(-):8				
	Urobilinogen	(+-)	Placebo	(+-):8				(+-):8					(+-):8					(+-):8				
			CP2305	(+-):8				(+-):8					(+-):8					(+-):8				
	Urinary bilirubin	(-)	Placebo	(-):8				(-):8					(-):8					(-):8				
			CP2305	(-):8				(-):8					(-):8					(-):8				
	Urinary ketones	(-)	Placebo	(-):8				(-):8					(-):8					(-):7 (+):1				
			CP2305	(-):7 (+):1				(-):8					(-):8					(-):8				
	Urine occult blood	(-)	Placebo	(-):7 (++):1				(-):5 (+-):2 (+):1					(-):6 (+-):2					(-):8				
			CP2305	(-):6 (+-):1 (+++):1				(-):5 (+-):2 (+):1					(-):7 (+++):1					(-):8				
	Urine ｐＨ	5.0～7.5	Placebo	6.13	±	0.64		6.13	±	0.92	NS		6.38	±	0.64	NS		6.31	±	0.80	NS	
			CP2305	6.44	±	0.73	NS	6.38	±	0.79	NS	NS	6.06	±	0.50	NS	NS	6.19	±	0.70	NS	NS
	Urine specific gravity	1.006～1.030	Placebo	1.0153	±	0.0069		1.0121	±	0.0080	NS		1.0129	±	0.0061	NS		1.0153	±	0.0078	NS	
			CP2305	1.0131	±	0.0095	NS	1.0136	±	0.0085	NS	NS	1.0106	±	0.0071	NS	NS	1.0119	±	0.0070	NS	NS

**：Significant difference compared to 0 W（p < 0.01）

*：Significant difference compared to 0 W（p < 0.05）

##：Significant difference between the groups（p < 0.01）

#：Significant difference between the groups（p < 0.05）

NS：No significant difference

†：The reference values are those from the time of testing (2016).

BA：Significance test in comparison with 0 W

BG：Testing for significance between groups

### 3.5 Medical questionnaire and daily report

Subjective symptoms reported during the study period are summarized in Table 7.

**Table 7:** List of subjective symptoms.

intaked food	Subject No.	Sex	Symptoms (background information, etc.) <Medication information>	Date of onset	Grade	Causal relationship with test food intake
Placebo	104	Male	Loose stools (disappeared after bowel movements around 10:00, possibly due to lack of sleep)	Day 1 and 2 of intake	Mild	With the exception of gas production, most of the symptoms were thought to be due to chronic fatigue or the effects of dietary content, but none of them were serious and were transient, or were observed in the follow-up observation period, so **there was no causal relationship with the intake of the test food.**
			Fatigue (resolved after bowel movements around 10:00, possibly due to lack of sleep)	Day 1 of intake	Mild	
			Gas (disappears after bowel movements, possibly due to fatigue)	Day 2 of intake	Mild	[Case excluded from analysis because the intake rate was below the specified value]
			Gas (maybe it was because of the ramen I had for lunch, but I'm not sure)	Day 6 of intake	Mild	
			Fatigue (all day, cause unknown)	Day 10 of intake	Mild	
			Stomach pain (due to gas buildup after ingestion. I went to the toilet several times before recovering. This may be because I was hungry and ate beef bowl before ingesting it)	Day 14 of intake	Mild	
			Urge to defecate (I had frequent urges in the morning and went to the toilet on about five floors in two hours. It wasn't diarrhea, but it was uncomfortable. The cause was unknown. The test food was not the cause. It got better in the afternoon.)	Day 15 of intake	Mild	
			Abdominal pain → diarrhea (may be caused by the milk at breakfast. No test food was ingested at this point)	Day 17 of intake	Mild	
			Fever (38°C fever, not severe stomach pain, but went to the toilet about 5 times in 2 h. Not diarrhea, but still uncomfortable. Probably due to cold symptoms)	Day 22 of intake	Mild	
			Gas buildup (I had gas buildup in the afternoon and had to go to the toilet several times. Each time it was relieved, but the discomfort continued.)	Day 24 of intake	Mild	
			Frequent gas (after going to bed. Feeling bloated.)	Day 25 of intake	Mild	
			Fatigue (I also had to defecate in the morning. I had not taken the test food at this point. I felt a little better after lunch, but the fatigue persisted. The cause is unknown, but it is thought to be because I had not yet fully recovered from my cold.)	Day 26 of intake	Mild	
			Urge to defecate (going to the toilet three times in the morning. Frequent urge to defecate but not diarrhea)	Day 27 of intake	Mild	
			Urge to defecate (I went to the toilet frequently in the morning. It wasn't diarrhea, but I felt uncomfortable. This may have been due to the effects of eating.)	Post-observation day 13	Mild	
			Stomach pain (the cause is unknown, but I haven't eaten anything unusual)	Post-observation day 15	Mild	
	114	Female	Headache (aftereffects of the accident, sometimes painful depending on the weather) <Taking headache medicine>	Day 19 of intake	Mild	Since the cause of the symptoms is known and the treatment is for that symptom, **there is no causal relationship with the intake of the test food.**
	115	Female	Stomach pain (maybe caused by eating dinner around 9 pm) <Take 1 packet of Pansilon>	Day 17 of intake	Mild	Since this is a symptom and treatment that may be affected by food, **there is no causal relationship with the intake of the test food**.
			Runny nose, sneezing, cough (hay fever) <Take Talion Tablets 10 mg and Pranlukast Tablets 225 "EK">	Post-observation day 10 & 11	Mild	This was a treatment for seasonal allergies, and **there was no causal relationship with the intake of the test food** due to the post-observation period.
	116	Female	Mouth ulcers (bite during dinner) <Take 2 tablets of Hythiol B>	Day 20 & 21 of intake	Mild	Since the cause of the symptoms is known and the treatment is for that symptom, **there is no causal relationship with the intake of the test food.**
	117	Female	Gas (I think it's because I drank this)	Day 2～25 of intake	Mild	Although the gas produced by the test food cannot be ruled out as a causal relationship, it is not a serious symptom and is not a problem. The other symptoms were transient and disappeared naturally without worsening, so **there is no causal relationship to the test food.**
			Belly rolling	Day 6 & 7 of intake	Mild	
			Loss of appetite, diarrhea, fatigue	Day 26 of intake	Mild	
			Difficulty passing stool	Day 27 of intake	Mild	
	122	Female	diarrhea	Day 2 of intake	Mild	It was determined that **there was no problem** because it was thought that the increase in fluid intake during excessive intake of the test food was related to the subject's habituation to the amount of fluid, and because this was a transient symptom that occurred when the subject experienced a defecation urge due to constipation.
			Abdominal pain (unknown cause, abdominal pain associated with defecation)	Day 2 & 3 of intake	Mild	
			abdominal bloating	Day 6 & 7 of intake	Mild	
CP2305	210	male	Slight diarrhea (due to eating too many vegetables)	Day 11 of intake	Mild	The cause of the symptoms is known, so **there is no causal relationship with the intake of the test food.**
	212	Female	Runny nose, cough, fatigue	Post-observation day 8～12	Mild	These were transient cold symptoms and were not a problem, with **no causal relationship to the intake of the test food**.
	214	Female	Headache (menstrual origin; symptoms present before ingestion)	Day 0 of intake	Mild	Since the symptoms were menstrual, there was no problem and **no causal relationship with the intake of the test food.**
			Nausea (menstrual origin; symptoms present before ingestion)	Day 1 of intake	Mild	
			Feeling tired? <Tiovita Drink>	Day 6, 16 & 27 of intake、	Mild	This was a transient symptom that was also observed during the follow-up period, and use of the drink to improve it was not a problem, and **there was no causal relationship with the intake of the test food.**
				Post-observation day 10		
			Feeling a bit sick? ≪Tiovita Drink≫	Day 9 of intake	Mild	The symptoms were transient, and the drink was used to improve the symptoms without any problems, and **there was no causal relationship with the intake of the test food.**
			Headache <Take one packet of Hedek Powder>	Day 8, 9 & 15 of intake	Mild	Although the cause is unknown, the symptoms were short-term and were resolved with medication, so there is no problem and **no causal relationship with the intake of the test food.**
			slight fever	Day 15, 19 & 25 of intake	Mild	This appears to be a temporary early symptom of a cold, so there is no problem and **no causal relationship with the intake of the test food.**
			fatigue	Day 16 of intake	Mild	This was a transient symptom that was also observed during the follow-up period, so there was no problem and **no causal relationship with the intake of the test food.**
				Post-observation day 10		
			Fatigue (menstrual origin)	Day 19 of intake	Mild	Since the symptoms were menstrual, there was no problem and **no causal relationship with the intake of the test food.**
			Sore throat (25th and 26th days after taking Benzablock L tablets)	Day 20, 21 & 25 of intake	Mild	This was due to transient cold symptoms that occurred from the end of the test food intake period through the post-observation period, and was treated accordingly, so there was no problem and **no causal relationship with the intake of the test food.**
			Runny nose (taking Benzablock S tablets)	Day 22 of intake	Mild	
			fever	Day 26 of intake	Mild	
			Cough (observation days 2 and 3 after taking Benzablock L tablets)	Post-observation day 10後観察2～5日目	Mild	
			phlegm	Post-observation day 10後観察2、3、6日目	Mild	
	215	Female	Abdominal pain (menstrual origin)	Day 18 & 19 of intake	Mild	Since the symptoms were menstrual, there was no problem and **no causal relationship with the intake of the test food.**
	216	Female	Hard stools (cause unknown)	Day 6, 25 & 26 of intake	Mild	The cause is unknown, and although **a connection to the test food cannot be denied,** it is a short-term symptom and is therefore not considered a problem.
			Difficulty passing stool (cause unknown)	Day 10 & 23 of intake	Mild	
	219	Female	Headache (possibly due to stiff shoulders or menstruation) <Taking Eve A tablets>	Day 21 of intake	Mild	Since the cause of the symptoms is known and the treatment is for that symptom, **there is no causal relationship with the intake of the test food**.
			Abdominal pain (menstrual origin) < <2nd day of observation after taking Eve A tablets> >	Day 28 of intake	Mild	Since the symptoms were menstrual, there was no problem and **there was no causal relationship with the intake of the test food** due to the observation period.
				Post-observation day 0 &2		
			Mild stomach pain (overeating)	Post-observation day 4	Mild	The cause of the symptoms is known, and **there is no causal relationship with the intake of the test food** because the condition is still in the observation period.
			Cough (Estac Ive, post-observation days 8–11)	Post-observation day 8～14	Mild	All of these were cold symptoms and treatments, and there was no problem. As this was a follow-up observation period, **there was no causal relationship with the intake of the test food.**
			snot	Post-observation day 10	Mild	
			headache	Post-observation day 12	Mild	
			Tired	Post-observation day 13	Mild	

In the placebo group, five subjects experienced one case each of headache, stomach pain, stomatitis, gas, stomach upset, loss of appetite, fatigue, difficulty in defecation, abdominal pain, or abdominal distension, as well as two cases of diarrhea during the study tablets ingestion period. During the follow-up period, one subject experienced a runny nose, sneezing, and cough. In the subgroup of constipated patients, diarrhea was observed in one male patient (#122) in the placebo group. In the CP2305 group, six subjects one case each of experienced mild diarrhea, nausea, cold-like symptoms, fatigue, sore throat, runny nose, hard stool, or difficulty in defecation, and two cases each of fatigue, headache, fever, and abdominal pain. During the follow-up period, one subject experienced phlegm, mild stomach pain, abdominal pain, and headache; two subjects reported runny nose and fatigue; and three subjects reported cough. One subject who was excluded from the analysis (male, No. 104 in the placebo group) also experienced symptoms similar to those of the other participants, such as gas and abdominal pain, both before and after ingestion of the study tablets. None of these symptoms were judged to be causally related to ingestion of the study tablets. Furthermore, no abnormal findings were observed during the physician’s medical examinations.

## 4 Discussion

This study investigated the safety of *L. gasseri* CP2305 tablets administered to healthy Japanese adults over a four-week period. Both groups received 20 tablets daily—either *L. gasseri* CP2305 tablets or placebo tablets. Safety was evaluated based on physical examinations, urinalysis, hematology, blood biochemistry, and subjective symptoms recorded in daily diaries.

In the physical examinations, no significant differences were observed between the groups in the overall analysis. In the subgroup analysis, systolic blood pressure was significantly higher in the CP2305 group at 2 W among healthy subjects. However, the mean value (125.8 ± 15.1 mmHg) remained below the reference threshold (<130 mmHg) and was therefore not considered clinically relevant from a safety standpoint. In addition, diastolic blood pressure was significantly higher in the placebo group among subjects prone to constipation; however, the mean value (84.1 ± 16.0 mmHg) was also below the reference threshold (<85 mmHg), indicating no safety concern. Although significant decreases in body weight and body fat percentage were observed before and after intake, these changes were small and showed no significant between-group differences, suggesting no safety implications. In addition, among healthy subjects, BMI showed a significant decrease in both the CP2305 and placebo groups. Because this change occurred similarly in both groups, it is considered to reflect seasonal or behavioral factors, such as natural fluctuations in daily activity levels, rather than any effect of the study tablets. A review of individual data revealed that a few participants had BMI or blood pressure values exceeding the reference limits; however, the number of such cases did not increase meaningfully after ingestion, indicating no effect attributable to the study tablets.

In hematological tests, no significant between-group differences were observed in either the overall or subgroup analyses. Although several parameters showed significant pre- to post-intake changes, all remained within normal ranges and were not considered clinically meaningful. Occasional outliers were observed, but the number of such cases did not increase notably after ingestion, indicating no influence of the study tablets.

In the blood biochemical tests, potassium levels were significantly higher in the CP2305 group at 6 W (post-observation period) in the overall analysis. In the subgroup analysis of healthy subjects, chloride at 2 W and potassium at 6 W were both significantly higher in the CP2305 group. In contrast, in subjects prone to constipation, potassium at 4 W and chloride at 6 W were significantly higher in the placebo group. Although several parameters showed significant pre- and post-intake changes, these variations were minor, transient, and within the normal range. They occurred in both groups, were not correlated with other related indicators, and were therefore not considered adverse effects associated with the study tablets.

Urinalysis revealed no significant between-group differences in quantitative parameters. Similarly, no notable between-group differences were observed in the number of subjects outside the reference limits for qualitative parameters. Although significant pre- and post-intake fluctuations were noted in urine specific gravity and pH, these changes were small and within the reference range, indicating no safety concern. A few subjects showed urinary occult blood levels of (±) or higher. But except for subject No. 111, who showed (±) result, all were female and likely influenced by menstruation. In subject No. 111, the result was deemed a false positive and transient, thus not considered clinically relevant.

Regarding subjective symptoms, a total of 14 events were reported by five subjects in the placebo group and 29 events by six subjects in the CP2305 group, including those reported during the post-observation period. All symptoms were mild, and no cases were considered to have a causal relationship with the study tablets, indicating that the product was safe. One excluded subject (No. 104, male, in the control group) reported abdominal symptoms during both the ingestion and post-observation periods; however, because these symptoms persisted even after ingestion ceased (up to day 15 of the post-observation period), a causal relationship with the study tablets was ruled out. In the subgroup of constipated patients, diarrhea was observed in one male patient (#122) in the placebo group, but no worsening of constipation was observed.

Overall, no safety concerns were identified in the physical examinations, hematology, blood biochemistry, urinalysis, or subjective symptoms. These findings confirm the safety of excessive intake of *L. gasseri* CP2305 tablets (10 times the recommended daily dose).

## 5 Conclusion

This study evaluated the safety of daily ingestion of 20 *Lactobacillus gasseri* CP2305 tablets (1.0 × 10 ¹¹ *L. gasseri* CP2305 cells/day) in healthy Japanese adults and individuals prone to constipation. No safety concerns were identified. These findings support the safety of *L. gasseri* CP2305 tablets, even when ingested at a dose ten times higher than the usual amount.

## Funder

Asahi Group Holdings Ltd.

## CRediT authorship contribution statement

**Kazuhiko Takano:** Supervision, Project administration, Investigation. **Hiroki Miyashita:** Writing – review & editing, Writing – original draft, Visualization, Validation, Software, Formal analysis, Data curation. **Tatsuhiko Hirota:** Writing – review & editing. **Koji Mochizuki:** Writing – review & editing, Supervision, Resources. **Daisuke Sawada:** Writing – review & editing. **Hajime Fuchida:** Writing – review & editing, Writing – original draft, Resources, Conceptualization.

## Declaration of Competing Interest

This study was sponsored by the following companies: Asahi Group Holdings, Ltd., which funded the study and its implementation, and Asahi Quality and Innovations, Ltd., which funded the manuscript preparation. H. F., K. M., D. S., and T. H. are employees of Asahi Quality and Innovations, Ltd. Asahi Group Holdings, Ltd., and Asahi Quality and Innovations, Ltd. entrusted the study operations to Clinical Support Corporation. H. M. is an employee of Clinical Support Corporation. Clinical Support Corporation was involved in the planning and implementation of the study as well as in manuscript preparation. Furthermore, K. T. (MD) served as the principal investigator and monitored all subjects’ conditions.

## Data Availability

Data will be made available on request.
